# The Role of GmSnRK1-GmNodH Module in Regulating Soybean Nodulation Capacity

**DOI:** 10.3390/ijms24021225

**Published:** 2023-01-08

**Authors:** Minglong Li, Yuye Wang, Pengmin Zhang, Chunxu Bai, Lei Cao, Ludan Li, Jihong Jiang, Xiaodong Ding, Jialei Xiao

**Affiliations:** 1 Key Laboratory of Agricultural Biological Functional Genes, Northeast Agricultural University, Harbin 150030, China; 2 School of Life Science, Jiangsu Normal University, Xuzhou 221116, China; 3 Key Laboratory of Soybean Biology of Chinese Education Ministry, Northeast Agricultural University, Harbin 150030, China

**Keywords:** soybean, nodulation, symbiosis, SnRK1, GmNodH

## Abstract

SnRK1 protein kinase plays hub roles in plant carbon and nitrogen metabolism. However, the function of SnRK1 in legume nodulation and symbiotic nitrogen fixation is still elusive. In this study, we identified GmNodH, a putative sulfotransferase, as an interacting protein of GmSnRK1 by yeast two-hybrid screen. The qRT-PCR assays indicate that *GmNodH* gene is highly expressed in soybean roots and could be induced by rhizobial infection and nitrate stress. Fluorescence microscopic analyses showed that GmNodH was colocalized with GsSnRK1 on plasma membrane. The physical interaction between GmNodH and GmSnRK1 was further verified by using split-luciferase complementary assay and pull-down approaches. In vitro phosphorylation assay showed that GmSnRK1 could phosphorylate GmNodH at Ser193. To dissect the function and genetic relationship of GmSnRK1 and GmNodH in soybean, we co-expressed the wild-type and mutated *GmSnRK1* and *GmNodH* genes in soybean hairy roots and found that co-expression of *GmSnRK1*/*GmNodH* genes significantly promoted soybean nodulation rates and the expression levels of nodulation-related *GmNF5α* and *GmNSP1* genes. Taken together, this study provides the first biological evidence that GmSnRK1 may interact with and phosphorylate GmNodH to synergistically regulate soybean nodulation.

## 1. Introduction

Legumes, members of the Fabaceae/Leguminosae family, are the third largest family of higher plants with almost 20,000 species belonging to 650 genera, and are ubiquitous all over the world [1]. Soybeans (*Glycine max*) are important economic crops that provide abundant and high-quality oil and protein for human beings [2]. Rhizobia aids soybean to fix nitrogen in the soil, allowing legumes to grow in poor soils deficient in mineral and organic nitrogen [3].

SNF1-related protein kinase 1 (SnRK1) is the plant orthologue of an evolutionarily conserved family of energy-sensing, central metabolic regulators that include yeast sucrose non-fermenting 1 (SNF1) and mammalian AMP-activated protein kinase (AMPK) [4]. The SnRK1 family kinases coordinate the energy and redox homeostasis of plants in response to a plethora of growth-inhibiting stresses and regulate a broad range of metabolic pathways through the phosphorylation of the key enzymes or transcription factors to improve stress tolerance and promote survival [5]. The protein exists as a complex formed by the SnRK1α catalytic subunit and the SnRK1β and SnRK1γ regulatory subunits. The roles of SnRK1 in restoring energy balance range from regulating metabolic processes, phosphorylation of key enzymes, to regulating significant changes in gene expression [6].

Nitrate reductase (NR), a key enzyme that catalyzes the first step of nitrate assimilation, was one of the first targets of SnRK1 discovered in plants. In spinach, phosphorylation of nitrate reductase by SnRK1 inhibited the in vitro activity of nitrate reductase [7]. In Arabidopsis, SnRK1 promotes the phosphorylation of NR1 and NR2, and negatively regulates NR activity [8]. Therefore, NR enzymes may be the main targets of SnRK1 in regulating nitrate assimilation in plants. In yeast and mammals, Snf1/AMPK activity is tightly regulated by cellular nitrogen levels. Activation of Snf1/AMPK under low nitrogen conditions inhibits TOR activity through direct phosphorylation-mediated inhibition of RAPTOR [9]. Under nitrogen sufficient condition, TORC1 inhibits Snf1 activity by downregulating Thr210 phosphorylation in the activation loop of the kinase subunit [10]. However, how TORC1 inhibits Snf1 activity remains to be determined in yeast. Recently, in mammals and *Saccharomyces cerevisiae*, TORC1 was found to directly inhibit AMPK signaling through phosphorylation of evolutionarily conserved serine residues in the AMPK kinase subunit [11]. Therefore, the interaction between TORC1 and Snf1/AMPK/SnRK1 may be critical for growth regulation according to nitrogen availability in eukaryotes. In Arabidopsis, nitrogen starvation moderately increased the in vitro activity of SnRK1 [12]. However, the physiological significance of SnRK1 signaling in nitrogen starvation is still unclear in plants.

Sulfate transferases/sulfotransferases (SOTs) are sulfate-regulated proteins in a variety of organisms. In plants, the binding reaction of sulfate plays a crucial role in the growth and development of plants and the response to various stresses [13]. Studies have shown that *SOT* genes can regulate plant stimuli responses, stress sensing and signaling mechanisms, and developmental processes. For example, in rice, the expression levels of 11 *OsSOTs* showed significant up- and downregulation in different tissues due to dehydration, high or low temperature, and hormonal stresses [14].

The symbiotic relationship between legumes and rhizobia typically begins in nitrogen-deficient soils, where legumes secrete a class of metabolites called flavonoids [15]. Rhizobia recognizes these signaling compound to trigger the synthesis and release of lipid oligosaccharide (LCoS)/nodulation (Nod) factors through nodulation genes/proteins of rhizobia [16]. Flavonoids in legume root exudates act as chemotactic signals for rhizobia under low nitrogen conditions, and together with isoflavones, they confer host specificity [17]. For example, isoflavones found in the root exudates of soybean and common bean induce the expression of nodulation genes in their compatible rhizobia symbionts, respectively [18]. Host-specific flavonoids are thought to interact directly with rhizobial NodD protein, a transcription factor (TF) that induces the expression of “normal” nodulation genes, resulting in the production of lipo-oligosaccharides with a conserved core structure Nodulation factors (NFs) [19]. Partner selection is further facilitated by various side groups generated by enzymes encoded by “host-specific” nodulation genes [20].

In this work, we determined the physical and genetic interaction of GmSnRK1 with the sulfotransferase GmNodH, validating that GmNodH is a phosphorylated substrate of the GmSnRK1 protein kinase. Their effects on the symbiotic relationship of soybean root nodules were discussed to provide some references for analyzing the molecular mechanisms of nodulation and symbiotic nitrogen fixation in legumes.

## 2. Results

### 2.1. Identification of GmNodH from Soybean

In our previous work [21], we screened a soybean cDNA library by using GmSnRK1 as bait by yeast two-hybrid approach and obtained a clone encoding GmNodH as a GmSnRK1-interacting protein. The bioinformatics analysis (ProtParam http://web.expasy.org/protparam/, accessed on 6 March 2021) showed that the soybean GmNodH consists of 341 amino acids, with a relative molecular mass of 39.7 kDa and an isoelectric point of 8.94. Prediction of GmNodH protein functional domain by Pfam (http://pfam.xfam.org/, accessed on 6 March 2021) and SMART (http://smart.embl-heidelberg.de/, accessed on 6 March 2021) suggests GmNodH has a putative sulfotransferase domain from the 99th to 322nd amino acid (Supplementary Figure S1).

**Figure 1:**
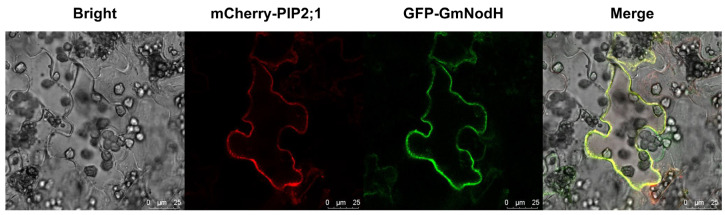
Subcellular localization of GmNodH protein. GFP-GmNodH is located in plasma membrane. mCherry-tagged PIP2;1 is a plasma membrane marker protein.

More prediction showed that GmNodH protein has a significant plasmamembrane localization signal (Supplementary Figure S2). To verify this prediction, we co-expressed GmNodH-GFP and PIP2;1-mCherry in the leaves of *Nicotiana benthamiana*. The results showed that GmNodH-GFP is overlapped with mCherry-tagged PIP2;1 which is a typical plasma membrane-localized protein (Figure 1).

### 2.2. Analysis of GmNodH Expression Patterns

**Figure 2:**
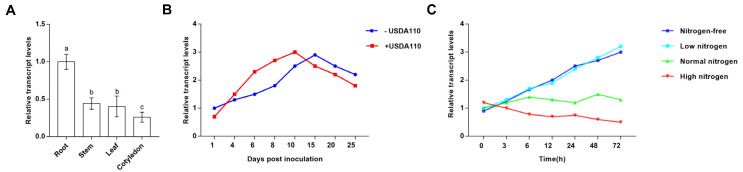
Analysis of *GmNodH* expression patterns. (**A**) The spatial expression patterns of *GmNodH* in different tissues of cultivated soybean. (**B**) Transcription levels of *GmNodH* in roots were induced by rhizobial treatments. (**C**) Response of *GmNodH* to different nitrate concentrations. The experiments were repeated three times, the data are shown as mean ± SD, the error lines are expressed as SE (*t* test), and different lowercase letters are used to show the difference in significance *p* < 0.05.

To investigate the physiological function of GmNodH, we measured the spatial expression patterns of *GmNodH* gene in soybean tissues (root, stem, leaf, cotyledon) by using qRT-PCR approach. We found that *GmNodH* gene was differentially expressed in soybean plants, in which *GmNodH* demonstrates the highest expression level in roots (Figure 2A). However, *GmNodH* did not show significant difference in root tissues and nodules (Supplementary Figure S3).

In order to determine whether the *GmNodH* gene can be induced by rhizobia, we inoculated the soybean plants with the slow-growing rhizobia USDA110, and sampled the infected plants after 1-, 4-, 6-, 8-, 10-, 15-, 20-, and 25-day treatment for qRT-PCR analyses. The results showed that the expression level of *GmNodH* was stimulated after rhizobia infection (4 days), and then rose and fell around 10 days (Figure 2B). The results indicated that *GmNodH* was responsive to rhizobia infection at the transcriptional level, suggesting that *GmNodH* may be involved in soybean nodulation and symbiotic nitrogen fixation.

Legumes can only establish a symbiotic relationship with rhizobia under low nitrogen conditions to form a special organ–root nodule [22]. Therefore, we tested whether the level of nitrate nitrogen can affect the expression of *GmNodH* gene. Therefore, we grew 4-week soybean plants in nitrogen-free, low nitrogen (3 mM NO_3_^−^), normal nitrogen (10 mM NO_3_^−^), and high nitrogen (20 mM NO_3_^−^) conditions for 0, 3, 6, 12, 24, 48, and 72 h [23], and sampled the treated plants for analyses of expression levels of *GmNodH* gene by qRT-PCR analyses. The results indicated that *GmNodH* could significantly induced by low nitrogen condition and was repressed by high nitrogen (Figure 2C). The responses of *GmNodH* to rhizobia and nitrate treatments suggest that *GmNodH* may be involved in soybean nodulation and nitrogen metabolism.

### 2.3. Overexpression of GmNodH Promotes Soybean Nodulation and Alters the Gene Expression Patterns in Nodulation-Related Pathways

**Figure 3:**
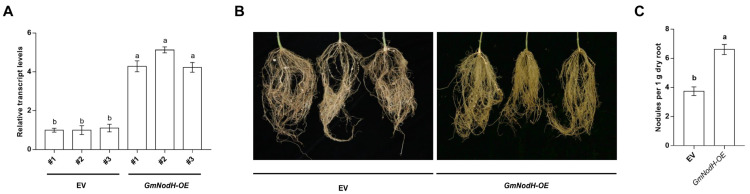

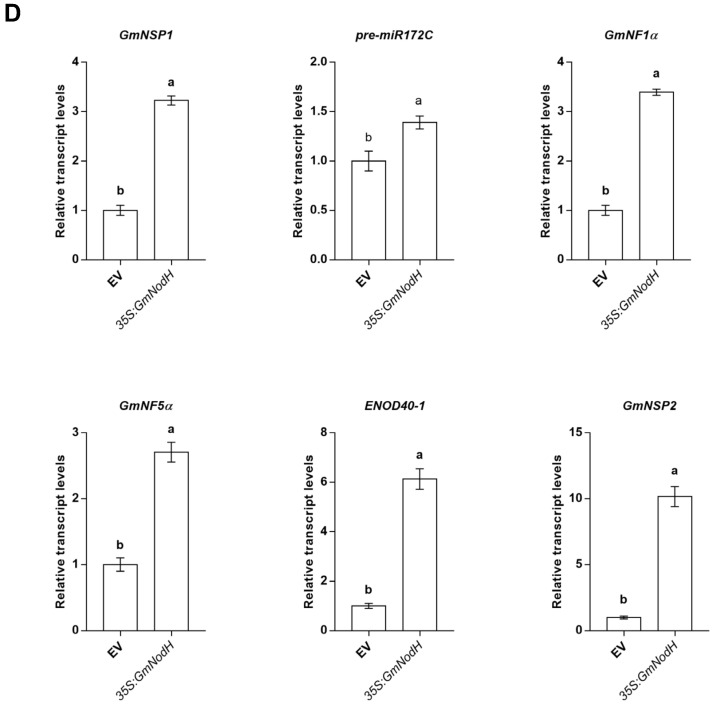
Overexpression of GmNodH promotes soybean nodulation and alters the expression of genes in nodulation-related pathways. (**A**) Expression levels of *GmNodH* in different transgenic soybean lines. (**B**) Overexpression of GmNodH promoted soybean nodulation. (**C**) Comparison of the number of nodules on the EV and GmNodH-overexpressing hairy roots 1-month after inoculation with rhizobia. The nodule numbers were normalized dry root weight (1 g). (**D**) Overexpression of GmNodH promoted the expression levels of nodulation-related genes such as *GmNF5α*, *pre-miR172C*, *GmNSP1*, *ENOD401*, *GmNSP2*, and *GmNF1α* in roots. The experiments were repeated three times, the data are shown as mean ± SD, the error lines are expressed as SE (*t* test), and the different lowercase letters are used to show the difference in significance *p* < 0.05.

To determine the effect of GmNodH on soybean nodulation, we constructed an overexpression vector haboring GmNodH gene driven by 35S promoter and transformed it into soybean hairy roots by *Agrobacterium rhizogenes*. The expression of *GmNodH* was confirmed by qRT-PCR in the transgenic hairy roots (Figure 3A). Afterwards, the composite plants were inoculated with Rhizobium USDA 110, and the plants were sampled for observation of nodulation after 1-month growth at normal condition. The results indicated that overexpression of GmNodH significantly increased the number of soybean hairy root nodules by approximately three-fold compared to EV controls (Figure 3B,C). Therefore, GmNodH may play a positive regulatory role in soybean nodulation.

Moreover, we determined whether overexpression of *GmNodH* altered the expression levels of genes in nodulation pathways by qRT-PCR. The results showed that overexpression of GmNodH promoted the expression levels of nodulation-related genes such as *GmNF5α*, *pre-miR172C*, *GmNSP1*, *ENOD401*, *GmNSP2*, and *GmNF1α* (Figure 3D).

### 2.4. Physical Interaction between GmSnRK1 and GmNodH

**Figure 4:**
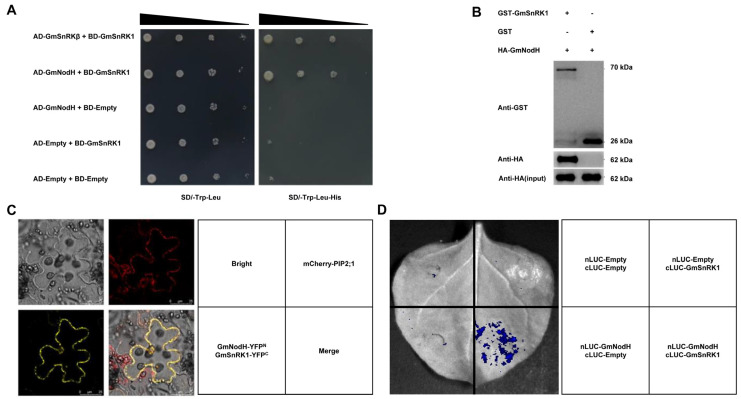
Physical interaction between GmSnRK1 and GmNodH. (**A**) Interaction of GmNodH and GmSnRK1 in yeast. (**B**) In vitro interaction of GmNodH and GmSnRK1 revealed by pull−down assay. (**C**) Analysis of interaction of GmNodH and GmSnRK1 by BiFC assay. (**D**) In planta interaction of GmNodH and GmSnRK1 by split−LUC complementation assays.

Since GmNodH was initially identified as a binding protein of GmSnRK1 from a screen of cDNA library, here, we verified their physical interaction by yeast two-hybrid experiment (Figure 4A). The pull-down assay indicated that the bait protein GmSnRK1 interacted with the target protein GmNodH in vitro (Figure 4B). Bimolecular fluorescence complementation (BiFC) experiments showed that a yellow fluorescent protein (YFP) signal generated by the interaction between GmSnRK1 and GmNodH was on the cell membrane and the YFP signal was overlapped with the fluorescence signal of PIP2;1-mCherry, suggesting that GmSnRK1 and GmNodH interacted and were co-localized on the plasma membrane (Figure 4C). When GmNodH-nLUC and GmSnRK1-cLUC were co-transformed into *N. benthamiana*, a significant LUC signal was detected (Figure 4D), confirming their physical interaction *in planta*.

### 2.5. In Vitro Phosphorylation Analysis of GmNodH by GmSnRK1

**Figure 5:**
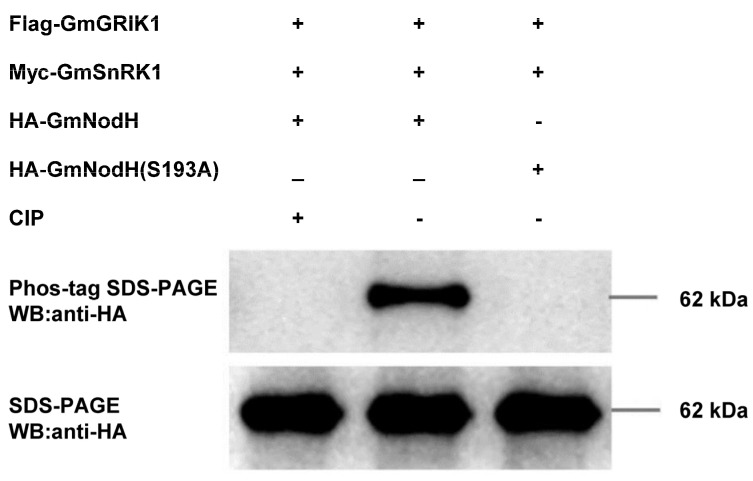
In vitro phosphorylation analysis of GmNodH by GmSnRK1 using Phos-tag technique.

We predicted seven potential SnRK1 phosphorylation sites on GmNodH by using the PPSP tool (http://ppsp.biocuckoo.org/, accessed on 12 November 2021). Of these putative phosphorylation sites, Ser193 has the highest score of 7.0 (Supplementary Figure S4). To verify these phosphorylation sites, we generated a series of GmNodH mutants and found that only S193A mutation could abolish phosphorylation of GmNodH by GmSnRK1. Treatment of phosphorylated GmNodH by calf intestine phosphatase (CIP) could eliminate the phosphorylation signal, confirming that GmNodH is a phosphorylation substrate of GmSnRK1 and Ser193 in GmNodH is the only phosphorylation site (Figure 5).

### 2.6. Coexpression of GmSnRK1 and GmNodH Can Promote Soybean Nodulation

**Figure 6:**
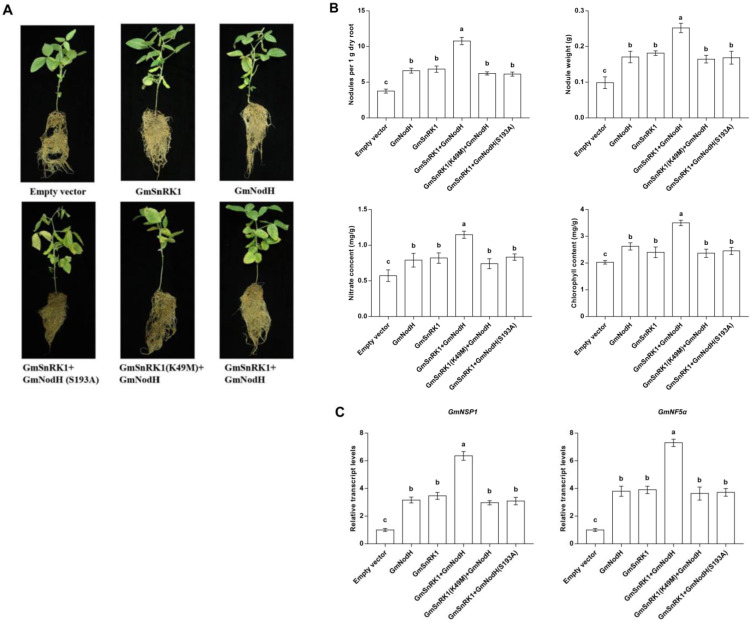
Phosphorylation of GmNodH by GmSnRK1 can promote soybean nodulation. (**A**) Phenotypic analysis of transgenic chimeric soybean. (**B**) Under rhizobial treatment, the phenotypic data of nodule number and nodule weight, and biochemical data of nitrate and chlorophyll contents. The nodule numbers were normalized dry root weight (1 g). (**C**) Expression patterns of GmNSP1 and GmNF5α genes in rhizobial-infected roots. The experiments were repeated three times, the data are expressed as mean ± SD, the error bars indicate SE (*t* test), and the different lowercase letters are used to indicate the significant difference *p* < 0.05.

To dissect the biological function and genetic relationship of GmSnRK1 and GmNodH in soybean, we co-expressed the wild-type and mutated GmSnRK1 and GmNodH genes in soybean hairy roots. The transgenic composite plants were equally inoculated with Rhizobium USDA110 and were grown for 30 days in the normal conditions. The plant hairy roots with different genetic background exhibited different nodulating abilities (Figure 6A). The phenotypic data showed that the plant hairy roots carrying GmSnRK1 and GmNodH bore the most nodules among the other composite plants. Compared to the EV control, single expression of GmSnRK1 or GmNodH and double expression of GmSnRK1 (K49M)/GmNodH or GmSnRK1/GmNodH (S193A) could also enhance nodulation rates.

Under rhizobial treatment, the phenotypic data of nodule number and nodule weight, and biochemical data of nitrate and chlorophyll contents of the co-expressed GmSnRK1/GmNodH transgenic chimera plants were significantly improved compared with the control groups, and the effect of positive regulation on soybean nodulation was more obvious (Figure 6B). The results implicate that the involvement of GmNodH in regulating soybean nodulation is achieved through the phosphorylation of GmSnRK1 protein kinase.

### 2.7. Expression Analyses of Genes Involved in Signaling Pathways of Nodulation

In order to further explore the effect of GmSnRK1-GmNodH module on signaling pathways, we analyzed the responses of genes involved in soybean nodulation signaling pathways. Since GmNF5α is a soybean nodulation factor gene and GmNSP1 is a transcription factor gene which are required for soybean nodulation initiation and nodulation [24], here, we investigated how GmSnRK1-GmNodH module regulated the expression levels of these two genes. The results showed that overexpression of GmSnRK1-GmNodH could significantly enhanced the expression levels of GmNF5α and GmNSP1 (Figure 6C), which is consistent to the phenotypes shown in Figure 6A.

## 3. Discussion

### 3.1. GmNodH Plays a Positive Regulatory Role in the Symbiotic Nitrogen Fixation of Soybean

In leguminous rhizobia symbiosis, the exchange of signals leads to the development of nodules, the special organ where rhizobia interact with the host plants to fix nitrogen [25]. The roots of a legume plant excrete flavonoid compounds to attract rhizobia, that, in turn, begin to produce the bacterial signal molecules called Nod factors (Nodulation factors) [26]. Specific plant flavonoids act as positive inducers of Nod gene transcription. The spatial structure of the nod gene promoter, the binding of RNA polymerase, and the initiation of Nod gene transcription were changed after the NodD protein of rhizobia was bound to specific flavonoids [19]. In *Rhizobium meliloti*, NodH factor determines host range by helping to mediate the production of a specific extracellular signal [27]. NodH catalyzes the transfer of sulfate from 3′-phosphoadenosine 5′-phosphosulfate to the terminal 6-O [28]. In alfalfa, the expression of nodulin gene MtENOD12 was significantly inhibited after inoculation with a nodH mutant compared to the wild-type *Rhizobium meliloti*, suggesting the sulfated Nod factors are required for nodulation by *R. meliloti* [29]. However, alfalfa nodulation by *Sinorhizobium fredii* does not required sulfated Nod-factors [30].

In plants, the binding reaction of sulfate plays a crucial role in the growth and development of plants and the response to various stresses [13]. The protein structure of GmNodH was in silico predicted to have a sulfotransferase domain (Figure S1). Sulfate/sulfotransferases (SOTs) are sulfate-regulated proteins in a variety of organisms. The presence of the sulfate modification on Nod factor is absolutely required for the establishment of the symbiosis of *Sinorhizobium meliloti* on alfalfa and is dependent on the products of three genes, nodH, nodP, and nodQ. The nodH gene product catalyzes the sulfonyltransfer to the Nod factor backbone, while the nodP and nodQ gene products form a sulfate-activating complex which catalyzes the conversion of sulfate to 3′-phosphoadenosine-5′-phosphosulfate, an activated form of sulfate used by all known carbohydrate sulfotransferases [31]. In *Lotus japonicus*, autophosphorylation is essential for the in vivo function of the *Lotus japonicus* Nod factor receptor 1 and receptor-mediated signaling in cooperation with Nod factor receptor 5 [32]. Recently, in woody plant Populus, a putative sulfotransferase PtSS1 gene was determined to be induced by treatment of lipo-chitooligosaccharides (LCOs) which are produced by rhizobia and a wide range of fungi, including mycorrhizal ones, and act as symbiotic signals that promote lateral root formation. PtSS1 gene can serve as a visual marker of LCO perception in Populus roots and can be used to study symbiotic interactions with the bacterial and fungal symbionts of Populus sp [33].

### 3.2. GmSnRK1 Regulates the Expression and Function of GmNodH Gene

The Snf1-Related Protein Kinase 1 (SnRK1) is the plant homolog of the heterotrimeric AMP-activated Protein Kinase/Sucrose Non-Fermenting 1 (AMPK/Snf1), which works as a major regulator of growth under nutrient-limiting conditions in eukaryotes [34]. SnRK1 is not only an important regulator of plant carbon metabolism, but also an important regulator of plant nitrogen metabolism. The fact that SnRK1 phosphorylates and inactivates nitrate reductase provides a route through which SnRK1 can affect nitrogen metabolism [35]. The regulation of nitrogen absorption is closely related to SnRK1 [8]. It has been reported that abundant SnRK1 and SnRK3-related protein kinases can be detected in the developing nodules of *L. japonicus* by immunological screening [36]. This means that GmSnRK1 may not only play an important role in plant energy, but also in nitrogen metabolism [37].

In this study, we determined that GmNodH gene expression levels altered to varying degrees after treating soybean seedlings with rhizobia and nitrate nitrogen stress. This result indicated that GmNodH was differentially expressed in soybean tissues and affected by rhizobia and nitrate nitrogen. Previously, GmSnRK1 was used as the bait protein to screen the soybean cDNA library by yeast two-hybrid approach, and two interacting proteins, GmNodH and GmNFR5α, were obtained. Both proteins are nodulation-related proteins, and GmNFR5α is a nodulation factor receptor [38]. It has been verified that GmSnRK1 can phosphorylate GmNodH, and it is speculated that GmSnRK1 can also phosphorylate GmNFR5α. In the future, we will determine whether GmSnRK1 may mediate the interaction of GmNodH and GmNFR5α to form a nodulation factor receptor complex to transduct the nodulation signals in soybean. In addition, when we co-expressed GmSnRK1/GmNodH genes, the expression levels of GmNF5α and GmNSP1 were significantly increased, providing more evidence that the GmSnRK1–GmNodH module may directly participate in regulating soybean nodulation.

## 4. Materials and Methods

### 4.1. Plant Materials and Growing Conditions

The soybean (*G. max* cv *Williams 82*) seeds were imbibed on moist filter paper at 28 °C in the dark for 48 h, and the seeds were germinated and grown under a 14-h photoperiod (200 μmol·m^−2^s^−1^ light intensity) at a constant temperature of 24 °C with a relative humidity of ~70%. The soybean seedlings were transferred to the garden soil at 26 °C and photoperiod of 16-h light/8-h dark.

### 4.2. RNA Extraction, cDNA Synthesis, and Quantitative Real-Time PCR

Plant total RNA samples were extracted using TRIzol reagent by following the manufacturer’s instructions. The Transcript ALL-in-One First-Strand cDNA Synthesis SuperMix kit (Transgen, Beijing, China) was used for cDNA syntheses. The TransStart Top Green qPCR Supermax kit (Transgene, Beijing, China) was used for qRT-PCR analysis. *GmGAPDH* was used as the reference gene for all qRT-PCR analyses. The 2^−ΔΔCT^ method was used to analyze the obtained data. The qRT-PCR primers for *GmNodH* and other stress-related genes are listed in Supplementary Table S1.

### 4.3. Subcellular Localization of GmNodH-GFP Protein

The pCAMBIA3301-*GmNodH*-*GFP* and pCAMBIA2300-*PIP2;1*-*mCherry* plasmids were transformed into *Agrobacterium tumefaciens* strain GV3101. The obtained bacterial strains were infiltrated into the abaxial air spaces of 4- to 6-week-old leaves of *N. benthamiana* plants by needleless syringe. After 48-h growth, the infected leaf sections were excised for observation of fluorescent signals under a confocal microscope.

### 4.4. Yeast Two-Hybrid Assay

For the Y2H assay, *GmSnRK1* and *GmNodH* genes were cloned into pGBKT7 and pGADT7 vectors, respectively. The combination of pGBKT7-*SnRK1β* and pGADT7-*SnRK1α* was used as a positive control. The obtained bait and prey constructs were co-transformed into Y2H Gold yeast competent cells.

### 4.5. BiFC and Split-LUC Complementation Assays

For BiFC analysis, the *GmNodH* and *GmSnRK1* coding sequences were subcloned into the *Sma*I and *Pma*I sites in pPBEL-BiFC vector, respectively [21]. The obtained constructs were introduced into strain GV3101 for transient expression in tobacco leaves. After 48-h growth, the infected leaf sections were excised for observation of fluorescent signals under a confocal microscope. For split-LUC complementation assays, the full-length *GmNodH* and *GmSnRK1* genes were cloned into pCAMBIA-1300-nLUC and pCAMBIA-1300-cLUC vectors, respectively, and co-infiltrated into tobacco leaves for LUC signals.

### 4.6. Protein Pulldown Assays

The GmNodH-HA and GmSnRK1-GST recombinant proteins were expressed and purified from *Escherichia coli.* GST-GmSnRK1 and GST alone were immobilized on GST resin and mixed with GmNodH-HA in the protein binding buffer (50 mM HEPES, 10% (*w*/*v*) glycerol, 1% (*w*/*v*) Triton X-100, 1.5 mM MgCl_2_, 150 mM NaCl, 1 mM egtazic acid, 1 mM phenylmethylsulfonyl fluoride, protease inhibitor cocktail, pH 7.5) with gentle agitation for 3 h at 4 °C. The immobilized proteins were washed for 5 times and were boiled for 10 min in 1× protein loading buffer. The protein samples were fractionated in 12.5% SDS-PAGE gels. The target proteins were detected by Western blotting using anti-HA or anti-GST antibodies (ABMART, Shanghai, China).

### 4.7. Protein Phosphorylation Assay

For in vitro phosphorylation analysis, the purified recombinant proteins (Flag-GmGRIK1, Myc-GmSnRK1, HA-GmNodH and their mutants) were added into kinase buffer (25 mM Tris (pH 7.4), 12 mM MgCl_2_, 1 mM dithiothreitol, and 1 mM ATP). The protein mixtures were incubated at 30 °C for 30 min. Dephosphorylation was carried out by treating the protein samples with calf intestine phosphatase (CIP). The reactions were stopped by addition of 1× protein loading buffer and boiled at 95 °C for 5 min. The protein samples were resolved in normal SDS-PAGE gel or SDS-PAGE gel with 12% acrylamide plus Zn^2+^-Phos-tag Biotin BTL-104 (NARD INSTITUTE, Ltd., 16A-03, Tokyo, Japan) according to the manufacturer’s instructions.

### 4.8. Transient Gene Expression and Induction of Hairy Roots in Soybean Plants

The wild-type *GmSnRK1*, *GmNodH* genes and their mutants were cloned into plant binary vectors. GmSnRK1 (K49M) is a kinase-dead mutant [21] and is used as a negative control. The constructs were transformed into *Agrobacterium rhizogenes* strain K599. The soybean (*Williams 82*) seeds were germinated in wet vermiculite and the seedlings were grown under conditions of 16-h light and 8-h dark photoperiod, 28 °C, and 80% soil moisture. When the cotyledons are fully opened and the shoots reach about 5 cm in height, a drop of *A. rhizogenes* culture (OD_600_ = 0.6–0.8) was injected into hypocotyls using a sterile syringe needle. The inoculated seedlings were covered with transparent Saran film to keep humidity and grown under the same conditions as above. After 15-day growth, the primary roots were removed from seedlings to induce the hairy roots when the hairy roots emerged. The plants with transgenic hairy roots are called composite plants.

### 4.9. Induction of Root Nodules

The composite plants with similar sizes of shoots and hairy roots were chosen for inoculation of rhizobia. The plant roots (30 plants for each group) were soaked in the diluted *Bradyrhizobium japonicum* USDA110 culture (OD_600_ = 0.01) or water for 10 min, and then the plants were transferred to the sterile soil for 1 week and were continued to grow with regular irrigation of nutrient solution under normal conditions for 30 days, and the phenotypic data of nodule number and nodule weight, and biochemical data of nitrate and chlorophyll contents were measured according to the described [39] and Grace Biotechnology manual.

### 4.10. Statistical Data Analysis

All experiments were carried out using biological duplications for each treatment and were replicated on at least three occasions. Multiple comparison tests were performed by Duncan’s test using SPSS statistical software (Version 16.0, SPSS). Significance level was set at *p* < 0.05.

## 5. Conclusions

We identified the physical and genetic interactions of GmSnRK1 with the sulfotransferase GmNodH, validated GmNodH as a phosphorylated substrate of GmSnRK1 protein kinase, and explored their effects on soybean root nodule symbiosis, providing the new evidence for dissecting the molecular mechanism of nodulation and symbiotic nitrogen fixation in soybean.

## Data Availability

Not applicable.

## Supplementary Materials

The supporting information can be downloaded at: https://www.mdpi.com/article/10.3390/ijms24021225/s1.
