# Supplementary material for: The Role of GmSnRK1-GmNodH Module in Regulating Soybean Nodulation Capacity
Source: Int J Mol Sci. 2023 Jan 8;24(2):1225. doi: 10.3390/ijms24021225 (PMC9861110; doi:10.3390/ijms24021225)
Supplement: Supplementary file 1 [file ijms-24-01225-s001.zip › Supplementary Figures.pdf]

Supplementary Figure S1

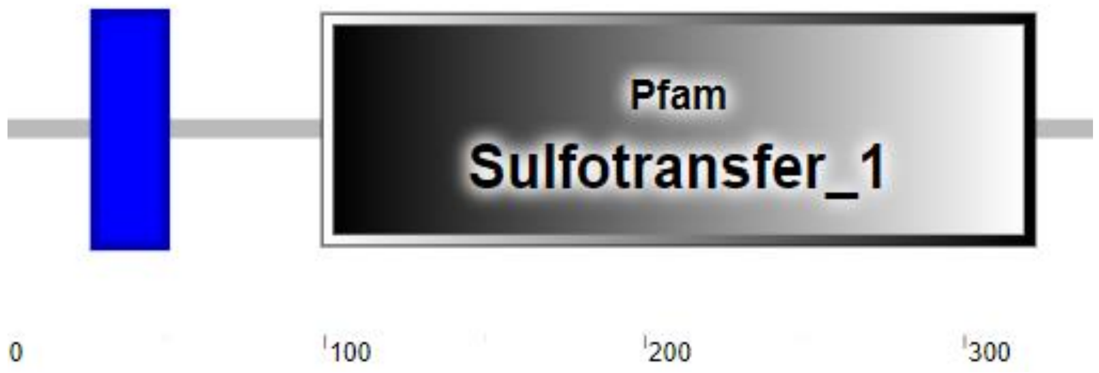

Supplementary Figure S2

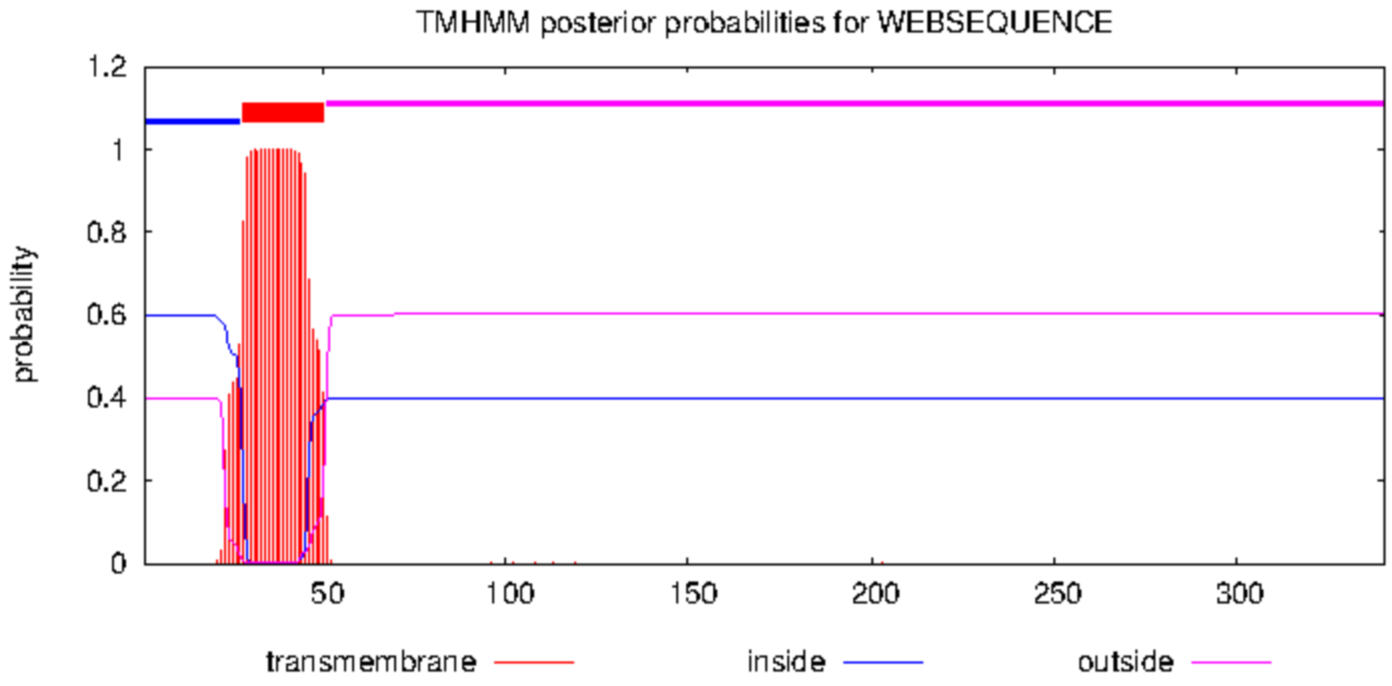

Supplementary Figure S3

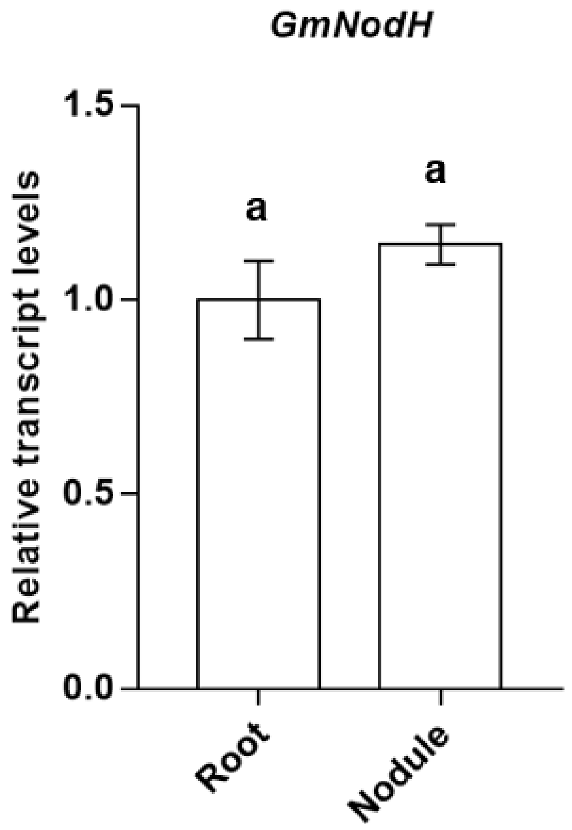

Supplementary Figure S4

Name	Position	Kinase	Peptide	Threshold	Risk-Diff.
	68	AMPK	NHSQSILKL	3.3	4.32
	85	AMPK	PKPSSFSRN	3.3	3.37
	107	AMPK	SNQRSGSGW	3.3	3.93
	109	AMPK	QRSGSGWFE	3.3	4.64
	139	AMPK	RQNVSSILL	3.3	5.08
	193	AMPK	HRRVSIIFL	3.3	7.00
	208	AMPK	RRMVSMLAN	3.3	5.33
